# Cysteine-rich protein 2 deficiency attenuates angiotensin II-induced abdominal aortic aneurysm formation in mice

**DOI:** 10.1186/s12929-022-00808-z

**Published:** 2022-04-12

**Authors:** Chung-Huang Chen, Hua-Hui Ho, Wei-Cheng Jiang, Wai-Sam Ao-Ieong, Jane Wang, Alexander N. Orekhov, Igor A. Sobenin, Matthew D. Layne, Shaw-Fang Yet

**Affiliations:** 1 Institute of Cellular and System Medicine, National Health Research Institutes, 35053 Zhunan, Taiwan; 2 Department of Chemical Engineering, National Tsing Hua University, 300044 Hsinchu, Taiwan; 3 Institute of Human Morphology, 117418 Moscow, Russia; 4 Laboratory of Medical Genetics, National Medical Research Center of Cardiology, 121552 Moscow, Russia; 5 Department of Biochemistry, Boston University School of Medicine, Boston, MA 02118 USA; 6 Graduate Institute of Biomedical Sciences, China Medical University, Taichung, 40402 Taiwan

**Keywords:** Abdominal aortic aneurysm, Cysteine-rich protein 2, Vascular smooth muscle cells, Collagen III, Matrix metalloproteinase 2

## Abstract

**Background:**

Abdominal aortic aneurysm (AAA) is a relatively common and often fatal condition. A major histopathological hallmark of AAA is the severe degeneration of aortic media with loss of vascular smooth muscle cells (VSMCs), which are the main source of extracellular matrix (ECM) proteins. VSMCs and ECM homeostasis are essential in maintaining structural integrity of the aorta. Cysteine-rich protein 2 (CRP2) is a VSMC-expressed protein; however, the role of CRP2 in AAA formation is unclear.

**Methods:**

To investigate the function of CRP2 in AAA formation, mice deficient in Apoe (Apoe^−/−^) or both CRP2 (gene name Csrp2) and Apoe (Csrp2^−/−^Apoe^−/−^) were subjected to an angiotensin II (Ang II) infusion model of AAA formation. Aortas were harvested at different time points and histological analysis was performed. Primary VSMCs were generated from Apoe^−/−^ and Csrp2^−/−^Apoe^−/−^ mouse aortas for in vitro mechanistic studies.

**Results:**

Loss of CRP2 attenuated Ang II-induced AAA incidence and severity, accompanied by preserved smooth muscle α-actin expression and reduced elastin degradation, matrix metalloproteinase 2 (MMP2) activity, deposition of collagen, particularly collagen III (Col III), aortic tensile strength, and blood pressure. CRP2 deficiency decreased the baseline MMP2 and Col III expression in VSMCs and mitigated Ang II-induced increases of MMP2 and Col III via blunting Erk1/2 signaling. Rescue experiments were performed by reintroducing CRP2 into Csrp2^−/−^Apoe^−/−^ VSMCs restored Ang II-induced Erk1/2 activation, MMP2 expression and activity, and Col III levels.

**Conclusions:**

Our results indicate that in response to Ang II stimulation, CRP2 deficiency maintains aortic VSMC density, ECM homeostasis, and structural integrity through Erk1/2–Col III and MMP2 axis and reduces AAA formation. Thus, targeting CRP2 provides a potential therapeutic strategy for AAA.

**Supplementary information:**

The online version contains supplementary material available at 10.1186/s12929-022-00808-z.

## Background

An aneurysm is a bulge or ballooning of a blood vessel. Abdominal aortic aneurysm (AAA), with the prevalence higher in smokers and in males [1], occurs in the abdominal aorta with a localized dilatation exceeding the normal diameter by more than 50%. Given the global average increase in age and lifespan [2], there is a significant concern of the rise of total AAA incidence and age-standardized mortality [3]. AAAs often grow slowly and are usually asymptomatic, making them difficult to detect until rupture, which has a high mortality rate of 85–90% [4]. One of the striking histopathological hallmarks in human AAA is the severe degeneration of the elastic media with extensive loss of elastin and vascular smooth muscle cells (VSMCs), resulting in decreased VSMC density, weakening of the aortic wall, and potential arterial wall rupture [5, 6]. The clinical approach to AAA is limited to surgical interventions due to lack of effective pharmacological treatments. Understanding the molecular mechanisms of AAA formation and progression is essential in identifying new therapeutic targets and devising pharmacological strategies for prevention and treatment of AAA disease.

The infrarenal abdominal aorta (IAA) is the most common site of human aortic aneurysm. Differential hemodynamic factors and regional features may explain the specific anatomic to site-specific degenerative changes [7]. Interestingly, a recent single-cell RNA sequencing study of IAA revealed cluster-specific, differentially expressed genes in the VSMCs. The top 5 genes were Myl9 (myosin light chain 9), Csrp2 (cysteine and glycine rich protein 2), Acta2 (smooth muscle (SM) actin α 2, SM α-actin), Tagln (transgelin, SM22α), and Rgs5 (Regulator of G protein signaling 5) [8], suggesting the potential importance of these VSMC genes in the development of AAA. Normally, VSMCs in the media display a differentiated, contractile phenotype and function to maintain vascular homeostasis [9]. Furthermore, VSMCs are a significant source of extracellular matrix (ECM) proteins, contributing to maintaining medial architecture and arterial structural integrity [10, 11]. In response to pathological stress, medial VSMCs increase ECM production resulting in adverse aortic wall matrix remodeling [11]. The phenotypic modulation of VSMCs is characterized by the downregulation of contractile genes and upregulation of matrix metalloproteinases (MMPs), which have been suggested to occur before aneurysm formation in animal models [12].

The Csrp2 gene encodes cysteine-rich protein (CRP) 2, a member of the LIM-only CRP family that contains two LIM domains [13]. In addition to cytoplasmic roles, CRP2 has been reported to form complex with serum response factor and GATA transcription factors in the nucleus to facilitate VSMC differentiation [14]. We have shown that CRP2 is preferentially expressed in VSMCs and downregulated by growth factor signaling and vascular injury [15, 16]. CRP2 interacts with actin crosslinking protein α-actinin, the adhesion plaque protein zyxin [13], and F-actin [17, 18]. In response to cellular migratory cues, the interaction of CRP2 with focal adhesion scaffold protein p130Cas attenuates lamellipodia formation, leading to reduced migratory ability of VSMCs, emphasizing additional cytoarchitectural function of CRP2 in VSMCs [19]. By gene deletion experiments in mice, we have previously shown that CRP2 deficiency increases neointima formation via enhanced VSMC migration into the intima following arterial injury [20], indicating important functions for CRP2 in occlusive vascular disease.

Gene expression profiling in human AAA and aortic occlusive disease (AOD) indicates that AAA and AOD have distinct pathogenic mechanisms [21]. For example, diabetes is a positive risk factor for AOD but is a negative risk factor for AAA [22]. CRP2 was found to be one of the top 5 VSMC cluster-specific, differentially expressed genes in IAA [8] and had protective functions in vascular occlusion [20]; however, whether CRP2 contributes to AAA development is unknown. In the current study, we used mice deficient in Apoe (Apoe^−/−^) or both Csrp2 and Apoe (Csrp2^−/−^Apoe^−/−^) in angiotensin II (Ang II)-induced AAA model to investigate the function of CRP2 in AAA formation.

## Materials and methods

### Animals

Csrp2-deficient (Csrp2^−/−^) mice on the C57BL/6 background generated previously [20] were crossed with Apoe^−/−^ mice (C57BL/6 background, Jackson Laboratory, Bar Harbor, ME, USA) to generate Csrp2^−/−^Apoe^−/−^ mice. Genotypes were confirmed by PCR. The primer pair (upper primer 5’-CCTGGGGCTTAGTGGTTTG-3’ and lower primer 5’-CCTGAGGAAAGAGTGACTAA-3’) was used for Csrp2 wild type allele of 563 bp; and the primer pair (upper primer 5’-TTGGGTGGAGAGGCTATTC-3’ and lower primer 5’-AGGAGCAAGGTGAGATGACA-3’) was used for Csrp2 knockout allele of 286 bp. For the Apoe allele, 3 primers were used in a PCR reaction to detect both wild type (155 bp) and knockout allele (245 bp) according to Jackson Laboratory’s protocol: 5’-GCCTAGCCGAGGGAGAGCCG-3’, 5’-TGTGACTTGGGAGCTCTGCAGC-3’, and 5’-GCCGCCCCGACTGCATCT-3’. Mice were housed in a specific pathogen-free animal facility at the National Health Research Institutes, Taiwan.

### Experimental mouse model of angiotensin II-induced AAA formation

All experimental procedures were performed in accordance with NIH guidelines for the care and use of laboratory animals and approved by the Institutional Animal Care and Use Committee of National Health Research Institutes, Taiwan (#NHRI-IACUC-104007-A). Approximately 12-week-old male Apoe^−/−^ and Csrp2^−/−^Apoe^−/−^ mice were subjected to an experimental AAA formation model [23, 24]. A Matrx VIP 3000 Vaporizer (Midmark, Miamisburg, OH, USA) was used to generate isoflurane vapor to anesthetize mice by inhalation, 4–5% initially and 1–3% during the procedure. Saline or Ang II (Sigma-Aldrich, St. Louis, MO, USA)-filled Alzet mini-osmotic pump (model 2004, DURECT, Cupertino, CA, USA) were implanted into the subcutaneous space in the back of the neck of the mouse and Ang II was infused at a rate of 1000 ng/kg/min. Following pump implantation, mice were fed a regular or high fat diet (D12108Ci; Research Diets, New Brunswick, NJ, USA) as described [25].

### Aortic tissue harvest, histological analysis, and immunohistochemistry

For fixation of aortic tissues, aortas were harvested at indicated time points following the Jackson Laboratory’s standard histology protocol (Jackson Laboratory). In brief, mice were deeply anesthetized with an overdose of tribromoethanol solution (500–750 mg/kg) by IP injection until they no longer displayed a withdrawal reflex in the hind limbs, and aortas harvested and processed for histological analysis. The maximal diameter of suprarenal aorta (SRA) and infrarenal aorta (IRA) were measured using calipers. Aneurysm formation was defined as the ratio of SRA/IRA ≥ 1.5. Plasma total cholesterol levels were measured 4 weeks after saline or Ang II infusion as described [25]. Paraffin-embedded abdominal aortic sections were stained with H&E for morphology, Masson’s trichrome (Sigma-Aldrich) for collagen, and Verhoeff’s stain (Sigma-Aldrich) for evaluating elastin degradation. For immunohistochemistry, the antibodies used are described in Additional file 1. For CRP2 and matrix metalloproteinase 2 (MMP2) staining, sections were treated with heated citrate buffer (ScyTek Laboratories, Logan, UT, USA) for 20 min, before blocking with 10% goat serum. Subsequently, sections were incubated overnight at 4 °C with CRP2 primary antibody, or room temperature for 1 h with MMP2 antibody. For matrix metalloproteinase 9 (MMP9), collagen type I (Col I), or collagen type III (Col III) immunohistochemistry, antigen retrieval was performed by placing sections in Trilogy buffer (Cell Marque, Rocklin, CA, USA) in a steamer for 20 min before blocking with antibody dilution buffer (Ventana Medical Systems, Tucson, AZ, USA). Sections were then incubated at room temperature for 1 h with primary antibody. To identify VSMCs, sections were stained for SM α-actin. Following primary antibody incubation, sections were incubated with Dako EnVision + System-HRP (horseradish peroxidase)-labelled polymer conjugated with secondary antibody (Agilent Technologies, Santa Clara, CA, USA). Dako liquid DAB + substrate chromogen system (Agilent Technologies) was subsequently used for the visualization of target proteins, followed by hematoxylin for counterstaining (Muto Pure Chemicals, Tokyo, Japan). Positive staining areas were quantified by colorimetric analysis using NIH ImageJ software and expressed as % positive area per section.

### Measurement of aortic reactive oxygen species (ROS) and ***in situ*** zymography

Abdominal aortas were isolated, embedded in OCT compound (Leica, Buffalo Grove, IL, USA), and frozen immediately. ROS levels of aneurysmal aortic wall sections (10 μm) were measured using fluorescent dye dihydroethidium (DHE, Molecular Probes, Eugene, OR, USA) as described [25]. *In situ* zymography was performed using EnzChek® gelatinase/collagenase assay kit (Molecular Probes) to measure MMP activity (including MMP2 and MMP9) according to the manufacturer’s protocol and as described [25]. Proteolytic activity was detected as green fluorescence. Digital images were captured on a BX51 microscope system (Olympus, Tokyo, Japan).

### Vascular smooth muscle cell culture and gelatin zymography assay

Primary VSMCs were prepared from adult or embryonic day 18.5 (or neonatal) Apoe^−/−^ and Csrp2^−/−^Apoe^−/−^ mouse aortas and cultured as described [19]. Cells of passages 5–7 were used for experiments. Cells were serum starved and then stimulated with Ang II (0, 1, and 10 µmol/L) for 24 h or indicated time periods and total proteins prepared for Western blot analysis to detect proteins of interest. To measure MMP2 and MMP9 activities by zymography, serum-starved VSMCs were stimulated with or without Ang II for 24 h, and the conditioned medium was collected and concentrated with Amicon® Ultra-15 centrifugal filter devices (MilliporeSigma, Burlington, MA, USA). Equal volumes of the concentrated medium were seperated by electrophoresis on 8% SDS-PAGE gels containing 0.8 mg/mL gelatin (Sigma-Aldrich). Gels were subsequently incubated with 2.7% Triton X-100 (Sigma-Aldrich) for 45 min at room temperature and then incubated with zymography developing buffer (50 mmol/L Tris, 0.2 mol/L HCl, 5 mmol/L CaCl2, 0.02% Brij35) at 37 °C for 36 h. Gels were then stained with PhastGel™ Blue R (GE Healthcare, Milwaukee, WI, USA). MMPs were detected according to their molecular weights and as clear bands against a blue background. The band intensities were quantified by Image J and normalized to control.

### Western blot analysis

Total proteins were prepared from adventitia-stripped aortas or VSMCs as described previously [20, 26] and subjected to SDS polyacrylamide gel electrophoresis. Proteins were then transferred to PVDF membranes (Merck Millipore, Danvers, MA, USA) for Western blot analysis. The antibodies used are described in Additional file 1. The blots were probed with primary antibodies to detect expression levels of CRP2, Col I, Col III, MMP2, and MMP9. To detect activation of p38 MAPK signaling pathways, blots were hybridized with antibodies for phosphorylated and total p38, Erk1/2, and JNK. In the rescue experiments, to confirm overexpression of CRP2-myc-His and CRP2-EGFP (enhanced green fluorescent protein), blots were probed with Myc and EGFP antibodies. Pan-actin or α-tubulin antibodies were used to verify equal loading. After primary antibody incubation, the blots were incubated with secondary antibody at room temperature for 2 h. Following secondary antibody incubation, blots were developed with chemiluminescent HRP substrate and then visualized by exposure to film. The protein bands were quantified by Image J and normalized to controls.

### Signaling pathway analysis

VSMCs were serum starved, stimulated with or without Ang II (10 µmol/L), and total proteins prepared for Western blot analysis to detect phosphorylated and total p38, Erk1/2, and JNK. To inhibit Erk1/2 activation, serum-starved VSMCs were pretreated with Erk1/2 inhibitor U0126 (InvivoGen, San Diego, CA, USA; tlrl-u0126) before stimulation with or without Ang II for 24 h. The conditioned medium was then collected for zymography to measure MMP2 activity whereas total proteins from the cell lysates were used for Western blot analysis to detect phosphorylated and total Erk1/2 and expression of MMP2, Col III, and CRP2. Vehicle controls without Ang II stimulation were set as 100%. Apoe^−/−^ and Csrp2^−/−^Apoe^−/−^ VSMCs were treated with Ang II for different time periods and the differences of Ang II-induced Erk1/2 activation were compared.

### Rescue of CRP2 expression in Csrp2^−/−^Apoe^−/−^ VSMCs

To reintroduce CRP2 expression in Csrp2^−/−^Apoe^−/−^ VSMCs, we used control (empty vectors) and 2 different CRP2 expression plasmids with either myc-His or EGFP tags [19]. Cells were electroporated with expression plasmids and serum starved for subsequent experiments. At early time points (0, 10, and 30 min) after Ang II stimulation, protein extracts were prepared to detect Myc, CRP2, and phosphorylated and total Erk1/2. At 24 h time point, the conditioned medium was collected for zymography to measure MMP2 activity and the cell lysates were collected for Western blot analysis.

### Measurement of systolic blood pressure and aortic tensile strength

A noninvasive tail-cuff method was used to measure systolic blood pressure (SBP) with a non-preheating MK-2000ST system (Muromachi Kikai, Tokyo, Japan). Conscious mice were restrained and acclimated in special mouse holders for 10 min before measurement. A minimum of 3 serial measurements was made and the average value calculated. The SBP of each mouse was measured at baseline before and at different time points after saline or Ang II infusion. To measure mechanical properties of the aortas, tensile test was performed using ElectroForce® 3200 Series III system (TA Instruments, Eden Prairie, MN, USA) as described [27]. Abdominal aortas were harvested from Apoe^−/−^ and Csrp2^−/−^Apoe^−/−^ mice with saline or Ang II infusion for 10 d. The abdominal aortas were extended in longitudinal direction at a constant extension rate of 0.025 mm/second with a 22.5 N load cell until failure and data were continuously collected as force vs. displacement curves. The ultimate tensile strength was calculated from the applied force upon specimen fracture.

### Statistical analysis

Percent incidence of AAAs between Apoe^−/−^ and Csrp2^−/−^Apoe^−/−^ mice was analyzed and compared by Fisher’s exact test. Student’s *t*-test was performed to compare AAA incidence and SRA/IRA ratio between Ang II-infused Apoe^−/−^ and Csrp2^−/−^Apoe^−/−^ mice. For elastin degradation grade and sample sizes with n = 3 or 4 per group, nonparametric Mann-Whitney test was performed to compare data sets between two groups. Two-way ANOVA was performed to compare data sets with two main factors (genotype and treatment), followed by Bonferroni’s post hoc test. Averaged values are presented as mean ± SEM. Statistical significance was recognized at *P* < 0.05.

## Results

### CRP2 expression in the abdominal aorta after Ang II infusion

**Fig. 1:**
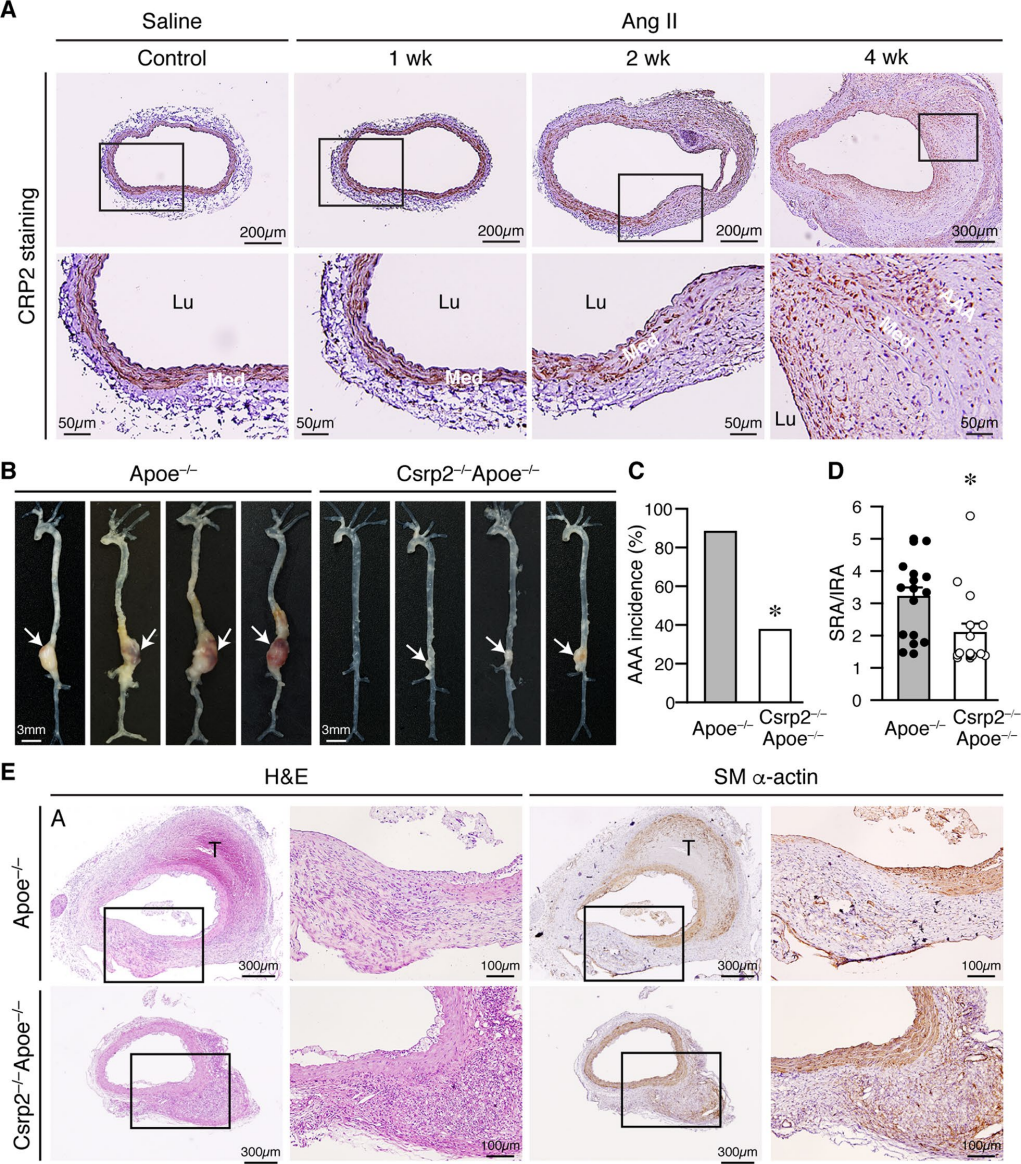
CRP2 deficiency protects against AAA formation. **A** Apoe^**−/−**^ mice were subjected to saline or Ang II infusion and abdominal aortas harvested at 1, 2, and 4 weeks for immunohistochemistry to detect CRP2 expression (brown). **B** Apoe^**−/−**^ and Csrp2^−/−^Apoe^**−/−**^ mice were infused with saline (Apoe^**−/−**^ n = 12, Csrp2^−/−^Apoe^**−/−**^ n = 13) or Ang II (Apoe^**−/−**^ n = 17, Csrp2^−/−^Apoe^**−/−**^ n = 16) mice were infused with saline for 4 weeks and representative aortas are shown. **C** Ang II infusion for 4 weeks induced AAA in 88% of Apoe^−/−^ mice (n = 17) while only 37.5% in Csrp2^−/−^Apoe^−/−^ mice developed AAA (n = 16). *P < 0.05 (Fisher’s exact test) compared with Ang II-infused Apoe^**−/−**^ group. **D** The ratio of SRA/IRA 4 weeks after saline or Ang II infusion in Apoe^−/−^ and Csrp2^−/−^Apoe^−/−^ mice. *P < 0.05 compared with the corresponding Ang II-infused Apoe^**−/−**^ group. **E** H&E staining and SM α-actin (brown) immunohistochemistry were performed on aneurysmal segments from Apoe^−/−^ and Csrp2^−/−^Apoe^−/−^ mice infused with Ang II for 4 weeks. Higher magnification of the boxed area is shown at the respective right panel. T, thrombus

To explore whether CRP2 has a role in AAA formation, we first examined the temporal expression pattern of CRP2 in the Apoe^−/−^ mouse abdominal aorta following Ang II infusion. Immunostaining revealed that CRP2 was strongly expressed in the medial smooth muscle layer of the aorta (Fig. 1A, saline control). CRP2 expression remained strong in the media 1 week after Ang II infusion and expression decreased in some areas of the media 2 weeks later (Fig. 1A, Ang II). At 4 weeks when AAA formation was apparent, CRP2 expression became patchy in the aneurysmal segment (Fig. 1 A, Ang II 4 wk). This expression pattern suggests that CRP2 may participate in AAA formation and progression, although it is not clear whether CRP2 reduction in the course of AAA development is harmful, or beneficial by functioning as an endogenous protective mechanism.

### Loss of CRP2 protects against Ang II-induced AAA formation

To determine the role of CRP2 in AAA formation, we generated Csrp2^−/−^Apoe^−/−^ mice and subjected Apoe^−/−^ and Csrp2^−/−^Apoe^−/−^ mice to the Ang II-induced AAA model and aorta examined after 4 weeks. Saline infusion did not induce aneurysm formation in either Apoe^−/−^ or Csrp2^−/−^Apoe^−/−^ mice (Additional file 2: Fig. S1A). After Ang II infusion, large AAAs were evident in Apoe^−/−^ mice; in comparison, AAAs were absent or smaller in Csrp2^−/−^Apoe^−/−^ mice (Fig. 1B). Compared with saline, Ang II notably increased AAA incidence and SRA/IRA ratio in both groups of mice; however, CRP2 deficiency in Apoe^−/−^ mice significantly reduced AAA incidence and SRA/IRA ratio, respectively (Fig. 1C, D). No differences in plasma cholesterol levels were detected after 4 weeks of saline or Ang II infusion (Additional file 2: Fig. S1B). Histological analysis revealed that there were no gross differences in vessel morphology, SM α-actin expression, or elastin layers between Apoe^−/−^ and Csrp2^−/−^Apoe^−/−^ mice 4 weeks after saline infusion (Additional file 2: Fig. S1C). However, infusion of Ang II for 4 weeks in Apoe^−/−^ mice induced AAA formation and thrombi were frequently observed (Fig. 1B, E). In contrast, Csrp2^−/−^Apoe^−/−^ mice had smaller aneurysms (Fig. 1B, E). Furthermore, compared with diminished SM α-actin expression in Apoe^−/−^ aneurysmal aorta, CRP2 deficiency resulted in preserved SM α-actin expression in the aortic media (Fig. 1E), indicating more VSMCs. These data indicate that CRP2 deficiency reduces aneurysm incidence and severity.

### CRP2 deficiency reduces elastin degradation, ROS level, and MMP activity

**Fig. 2:**
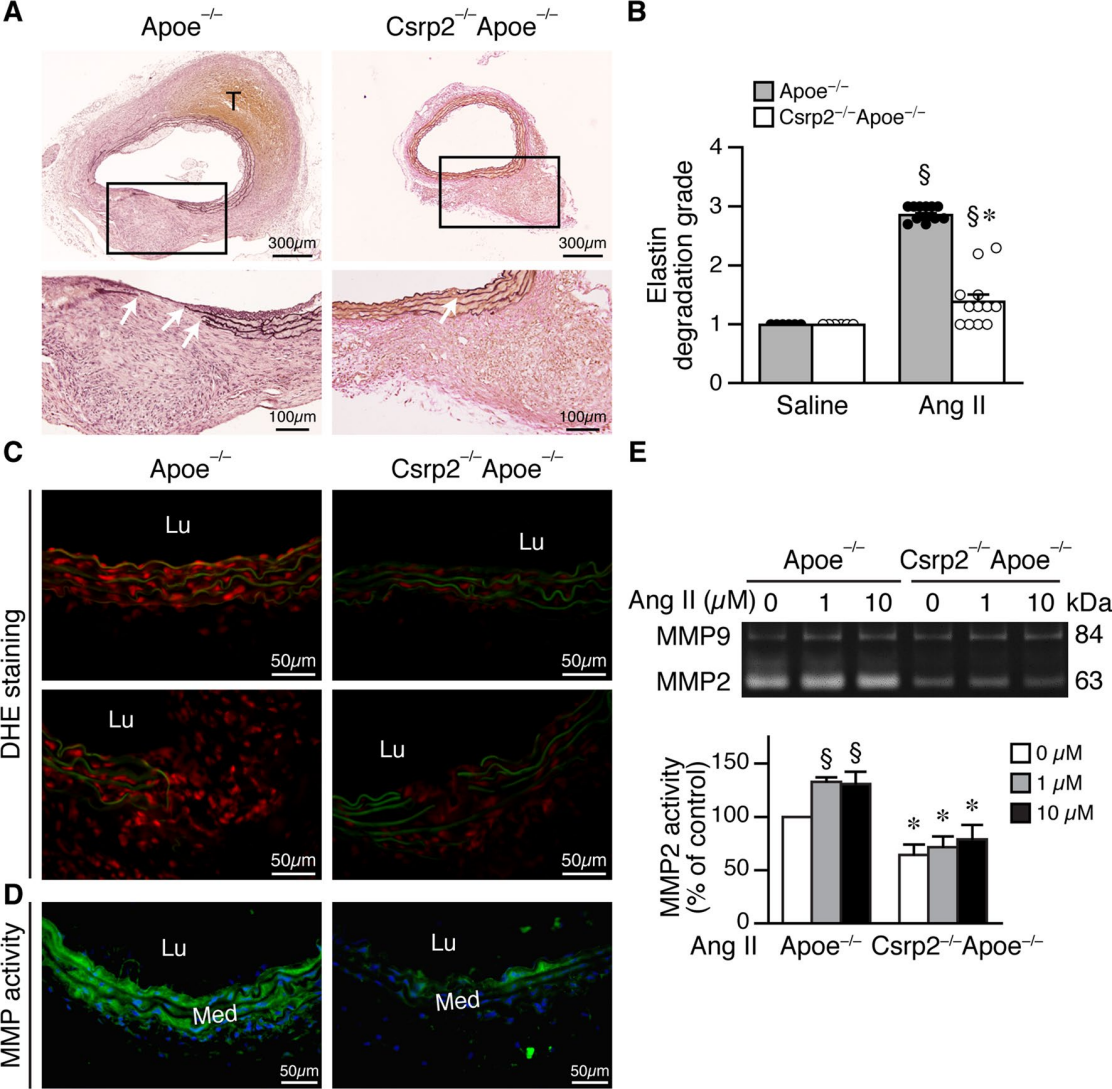
Loss of CRP2 reduces aortic elastin degradation and MMP activity. **A** Mice were infused with saline or Ang II for 4 weeks and abdominal aortas harvested for histological analysis. Upper panels, elastin staining of aneurysmal segments from Apoe^**−/−**^ and Csrp2^−/−^Apoe^−/−^ mice. Lower panels, higher magnification of the boxed areas from top row, respectively. White arrows indicate disrupted elastin fibers. **B** Quantitation of elastin degradation from Apoe^**−/−**^ mice infused with saline (n = 6) or Ang II (n = 12) and Csrp2^−/−^Apoe^−/−^ mice infused with saline (n = 6) or Ang II (n = 12). §*P* < 0.05 compared with the respective saline group; *P < 0.05 compared with the corresponding Ang II-treated Apoe^−/−^ group. **C** Mice were infused with Ang II for 2 weeks and DHE staining was performed to assess ROS level. **D** MMP activity (green fluorescence) was measured by *in situ* zymography from Apoe^**−/−**^ or Csrp2^−/−^Apoe^−/−^ mice infused with Ang II for 2 weeks. **E** Primary Apoe^**−/−**^ and Csrp2^−/−^Apoe^−/−^ VSMCs were stimulated with different concentrations of Ang II for 24 h and conditioned medium collected for zymography to examine MMP2 MMP2 and MMP9 activity. A representative zymography is shown in the upper panel. Quantitative analysis of MMP2 activity is shown in the lower panel (n = 4 each group). §*P* < 0.05 compared with the baseline without Ang II; *P < 0.05 compared with the corresponding Apoe^−/−^ group

Elastin degradation is one of the hallmarks of aneurysm formation [28], we next examined integrity of elastin layers 4 weeks after Ang II administration. Verhoeff’s elastin staining revealed that saline infusion did not cause any breakage of elastin layers in either group of mice (Additional file 2: Fig. S1C). Ang II caused increased elastin breakage in Apoe^−/−^ compared with Csrp2^−/−^Apoe^−/−^ mice (Fig. 2A). Csrp2^−/−^Apoe^−/−^ mice had a significantly lower elastin degradation grade (Fig. 2B). Because ROS contribute to AAA formation, we measured ROS level in the aortic wall 2 weeks after Ang II infusion. DHE staining showed that saline-infused mice exhibited faint red staining, indicative of low ROS levels (Additional file 2: Fig. S2A). In contrast, Ang II induced intense red staining, indicative of high ROS levels, in the aortic wall of Apoe^−/−^ mice (Fig. 2C). Interestingly, the DHE staining intensity was reduced in Csrp2^−/−^Apoe^−/−^ mouse aortic wall (Fig. 2C). MMPs are known to contribute to elastin degradation. Assessment of aortic MMP activity by *in situ* zymography revealed that compared with saline (Additional file 2: Fig. S2B), Ang II increased aortic wall MMP activity (green fluorescence) in both groups of mice; however, MMP activity was substantially lower in Csrp2^−/−^Apoe^−/−^ than in Apoe^−/−^ mice (Fig. 2D). MMP2 is a major MMP in VSMCs and degrades elastin and fibrillar collagen. Interestingly, patients with AAA have elevated MMP2 levels in the vasculature remote from the aorta, which is due to increased MMP2 expression from VSMCs [29]. This finding supports the systemic nature of aneurysmal disease and a primary role of MMP2 in aneurysm formation [29]. To further determine whether an absence of CRP2 contributed to reduced MMP activity, we treated primary VSMCs with Ang II for 24 h and measured MMP activity. VSMCs possessed strong MMP2 activity while MMP9 activity was rather weak at baseline (Fig. 2E). Interestingly, MMP2 activity was significantly lower in Csrp2^−/−^Apoe^−/−^ than that in Apoe^−/−^ VSMCs at baseline (Fig. 2E). Although Ang II increased MMP2 activity in VSMCs of both genotypes, the increase was significantly reduced in Csrp2^−/−^Apoe^−/−^ VSMCs (Fig. 2E). Loss of CRP2 did not appear to affect MMP9 in VSMCs (Fig. 2E). These results indicate that in response to Ang II stimulation, CRP2 deficiency attenuates Ang II-increased MMP2 activity in VSMCs and decreases ROS levels and elastin breakage in the media layer of the vessel, leading to decreased AAA formation.

### Absence of CRP2 reduces MMP2 expression in the mouse aorta

**Fig. 3:**
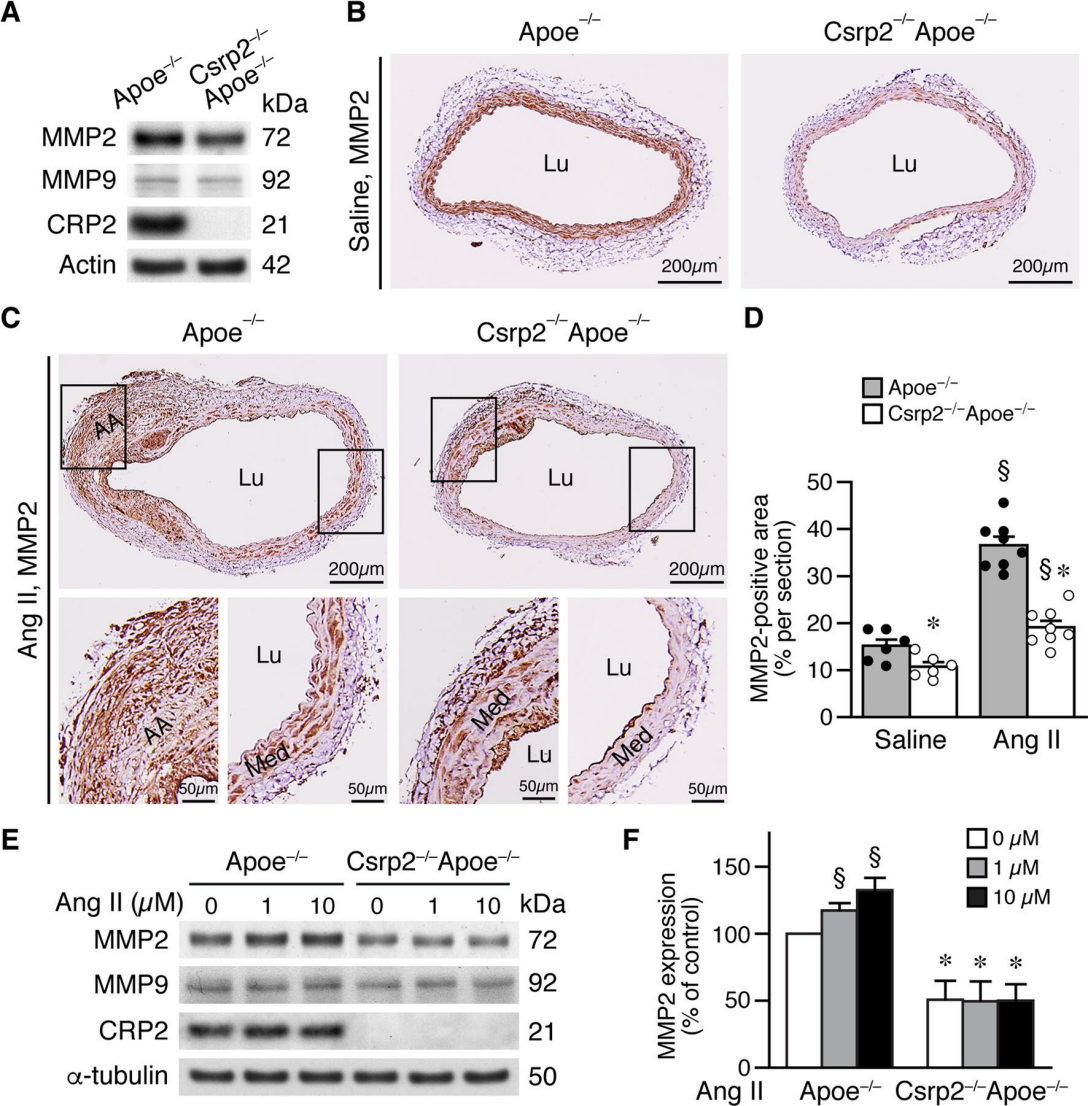
Absence of CRP2 reduces Ang II-induced MMP2 expression. **A** Protein extracts were prepared from Apoe^−/−^ and Csrp2^−/−^Apoe^−/−^ aortas and subjectd to Western blot analysis to detect MMP2, MMP9, and CRP2 expression. The blots were subsequently hybridized with actin as a loading control. **B** Mice were infused with saline or Ang II for 2 weeks and abdominal aortas harvested for MMP2 immunohistochemistry. Lu, lumen. **C** MMP2 immunostaining of aneurysmal sections from Apoe^**−/−**^ (n = 8) and Csrp2^−/−^Apoe^−/−^ (n = 8) mice infused with Ang II for 2 weeks. AA, aortic aneurysm; Med, media; Lu, lumen. Lower panels, higher magnification of the left and right boxed areas in the upper panels, respectively. **D** Quantification of MMP2-positive area in saline (n = 6 each group) and Ang II-infused (n = 8 each group) aortas. §*P* < 0.05 compared with the respective saline; *P < 0.05 compared with the corresponding Apoe^−/−^ group. **E** Apoe^**−/−**^ and Csrp2^−/−^Apoe^−/−^ VSMCs were stimulated with different concentrations of Ang II for 24 h and total proteins prepared for Western blot analysis to detect expression of MMP2, MMP9, and CRP2. The blots were subsequently hybridized with α-tubulin as a loading control. **F** Quantitation of MMP2 expression (n = 4 for Apoe^**−/−**^ and n = 3 for Csrp2^−/−^Apoe^−/−^ at each Ang II concentration). MMP2 levels in Apoe^−/−^ VSMCs without Ang II treatment was set as 100%. §*P* < 0.05 compared with the baseline without Ang II; *P < 0.05 compared with the corresponding Apoe^−/−^ group

To investigate the mechanisms underlying decreased MMP2 activity in Csrp2^−/−^Apoe^−/−^ mice, we prepared Apoe^−/−^ and Csrp2^−/−^Apoe^−/−^ mouse aortic protein samples and examined MMP levels by Western blot analysis. In comparison with Apoe^−/−^, Csrp2^−/−^Apoe^−/−^ aortas exhibited lower MMP2 level (Fig. 3A). Consistent with zymography result, MMP9 levels were low in both genotypes, and were not affected by loss of CRP2 (Fig. 3A). We next examined the expression of MMPs in the abdominal aorta by immunohistochemistry. Immunostaining showed that 2 weeks after saline infusion, aortic MMP2 levels were higher in Apoe^−/−^ than in Csrp2^−/−^Apoe^−/−^ mice (Fig. 3B) whereas MMP9 expression was barely detectable in both genotypes (Additional file 2: Fig. S3A). Ang II significantly induced MMP2 expression in the media and aneurysmal region of Apoe^−/−^ mice (Fig. 3C). In contrast, the induction of MMP2 expression in Csrp2^−/−^Apoe^−/−^ aortas by Ang II was less prominent (Fig. 3C). Quantitative analysis further confirmed that loss of CRP2 resulted in decreased MMP2 levels in either saline or Ang II-treated mouse aortas (Fig. 3D). We then stimulated VSMCs with or without Ang II and measured MMP2 and MMP9 expression levels. Consistent with aortic MMP2 expression (Fig. 3A), CRP2 deficiency lowered baseline MMP2 but not MMP9 levels (Fig. 3E). In comparison with increased MMP2 expression following Ang II stimulation in Apoe^−/−^ VSMCs, loss of CRP2 had attenuated response to Ang II on MMP2 induction but not MMP9 (Fig. 3E). Quantitative analysis showed the significant reduction of MMP2 in Csrp2^−/−^Apoe^−/−^ VSMCs (Fig. 3F). These results demonstrate that CRP2 deficiency reduces baseline and Ang II-induced MMP2 activity and expression level in VSMCs (Figs. 2 and 3).

### Lack of CRP2 attenuates Ang II-increased blood pressure and aortic tensile strength

**Fig. 4:**
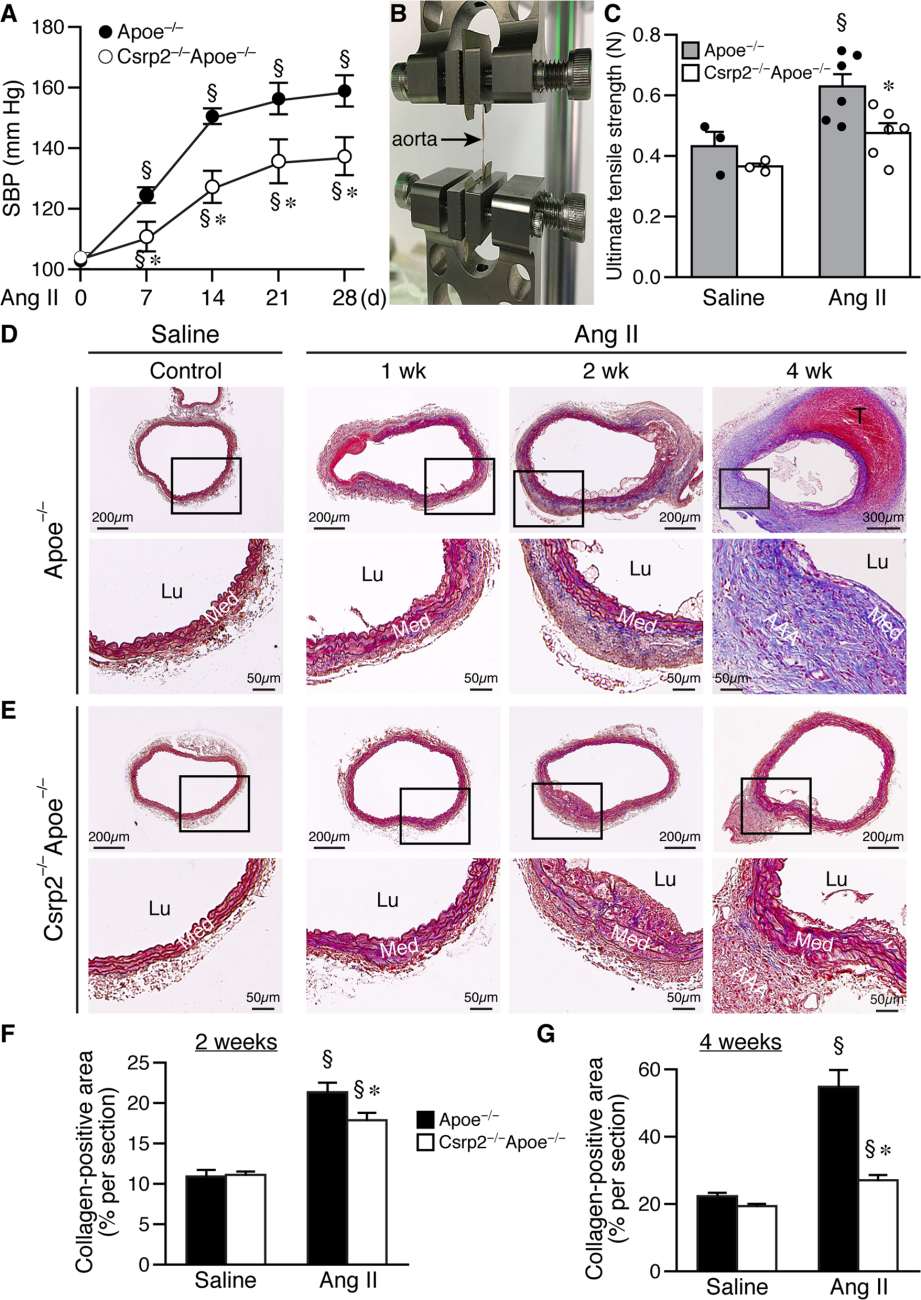
Loss of CRP2 attenuates Ang II-increased blood pressure and collagen deposition in aneurysmal mouse aorta. **A** Apoe^**−/−**^ (n = 13) and Csrp2^−/−^Apoe^−/−^ (n = 8) mice were infused with Ang II and SBP (mmHg) measured at baseline and at different days (7, 14, 21, and 28 d). ^§^*P* < 0.05 compared with the respective baseline at d 0; *P < 0.05 compared with the corresponding Apoe^−/−^ group. **B** Mice were infused with saline or Ang II for 10 d and abdominal aortas harvested for measurement of tensile strength using TA-ElectroForce® 3200 Series III system. ^§^*P* < 0.05 compared with saline group; *P < 0.05 compared with the corresponding Apoe^−/−^ group. **C** Quantitative analysis of the ultimate tensile strength of abdominal aortas from Apoe^**−/−**^ and Csrp2^−/−^Apoe^−/−^ (saline n = 3 each group, Ang II n = 6 each group). *P < 0.05. **D, E** Mice were infused with saline or Ang II and abdominal aortas harvested at different time points for histological analysis. Masson’s trichrome staining for collagen (blue) of aneurysmal segments from saline and Ang II-infused Apoe^**−/−**^ **D** and Csrp2^−/−^Apoe^−/−^ **E** mice at 1, 2, and 4 weeks. **D, E** Lower panels, higher magnification of the boxed areas in the respective upper panels. **F**, **G** Quantitative analysis of collagen-positive area of aneurysmal sections 2 weeks (**F**) and 4 weeks (**G**) after saline or Ang II infusion. Saline, n = 6 each group; Ang II, n = 8 each group. ^§^*P* < 0.05 compared with the respective saline group; *P < 0.05 compared with the corresponding Apoe^−/−^ group

Since hypertension is an identified risk factor for AAA [30], we next investigated whether CRP2 affected SBP. The two groups of mice had similar SBP at baseline (Fig. 4A). Although Ang II increased SBP during the 4-week period of AAA progression in both groups of mice, lack of CRP2 attenuated Ang II-increased SBP (Fig. 4A). Since arterial stiffness affects blood pressure [31, 32], we measured abdominal aortic tensile strength 10 d after saline or Ang II infusion (Fig. 4B). In the saline-infused mice, Csrp2^−/−^Apoe^−/−^ aortas exhibited slightly lower tensile strength than Apoe^−/−^ aortas, although the differences did not reach a statistical significance (Fig. 4C). Ang II infusion increased aortic ultimate tensile strength in both groups of mice (Fig. 4C), suggesting the aortas had become stiffer. Intriguingly, the Ang II-increased ultimate tensile strength in Csrp2^−/−^Apoe^−/−^ aortas was reduced compared to that of Apoe^−/−^ aortas (Fig. 4C), indicating a mitigated response to Ang II in the absence of CRP2. The mice in the AAA model described above were fed high-fat diet. To exclude the possibility that CRP2 affected AAA development through a secondary mechanism via high-fat diet, mice were infused with Ang II for 4 weeks and fed a regular chow diet. Ang II induced AAA in Apoe^−/−^ mice at a rate of 33.3% (Additional file 2: Fig. S4A, C), which was lower than that on high-fat diet (88.2%, Fig. 1C). No AAA was observed in Csrp2^−/−^Apoe^−/−^ mice (Additional file 2: Fig. S4B, C). With regular chow diet, Ang II progressively increased SBP during the 3-week period (Additional file 2: Fig. S4D). Importantly, CRP2 deficiency mitigated Ang II-increased SBP (Additional file 2: Fig. S4D), consistent with the observations when mice were fed high-fat diet.

### Loss of CRP2 reduces collagen deposition in aneurysmal mouse aorta

**Fig. 5:**
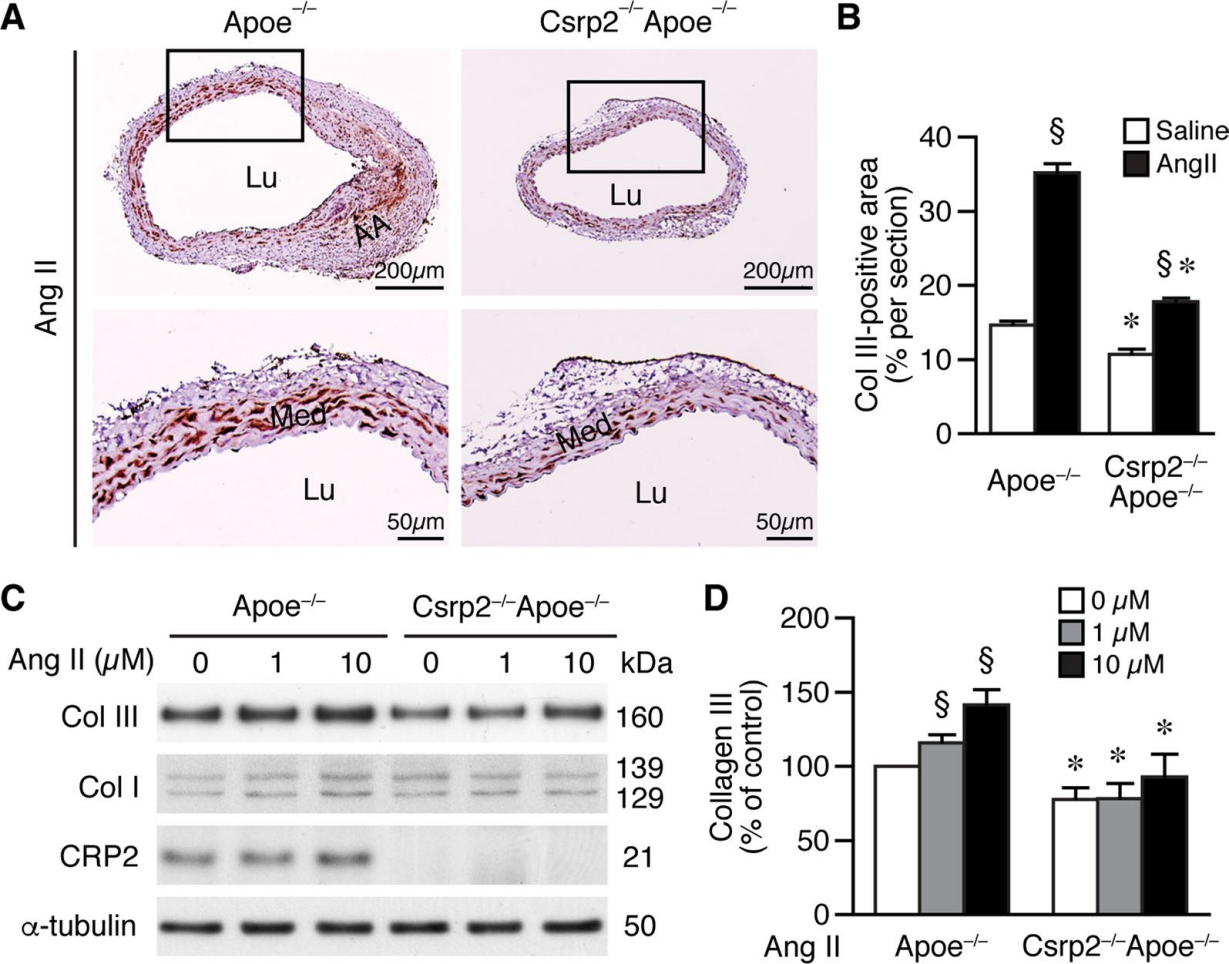
Lack of CRP2 reduces collagen III levels in vivo and in vitro. Mice were infused with saline or Ang II for 2 weeks and abdominal aortas harvested for immunohistochemistry. **A** Col III immunostaining of aneurysmal segment from Ang II-infused Apoe^**−/−**^ and Csrp2^−/−^Apoe^−/−^mice. Lower panels, higher magnification of the boxed areas in the upper panels, respectively. AA, aortic aneurysm; Lu, lumen; Med, media. **B** Quantitative analysis of Col III-positive area of aneurysmal Sect. 2 weeks after saline or Ang II infusion. Saline, n = 6 each group; Ang II, n = 8 each group. ^§^*P* < 0.05 compared with the respective saline group; *P < 0.05 compared with the corresponding Apoe^−/−^ group. **C** Apoe^**−/−**^ and Csrp2^−/−^Apoe^−/−^ VSMCs were stimulated with different concentrations of Ang II for 24 h and total proteins were prepared for Western blot analysis to detect Col III and CRP2 expression. The blots were subsequently hybridized with α-tubulin as a loading control. **D** Quantitation of Col III expression (n = 4 each group). Col III level of Apoe^−/−^ VSMCs without Ang II treatment was set as 100%. ^§^*P* < 0.05 compared with the baseline without Ang II; *P < 0.05 compared with the corresponding Apoe^−/−^ group

Collagen and elastin are the most abundant extracellular components of the aortic wall and the fibrillar collagen network contributes to aortic tensile strength, providing structural and mechanical support of the aorta [33]. However, increased collagen can enhance arterial stiffness, SBP elevation, and susceptibility to dissection and rupture [11]. Masson’s trichrome staining showed that basal collagen levels were low and similar between saline-infused Apoe^−/−^ and Csrp2^−/−^Apoe^−/−^ mouse aortas (Fig. 4D, E). Consistent with the report that collagen content is increased in the aneurysmal aorta [34], collagen levels progressively increased in Apoe^−/−^ mice during the development of AAA starting from 1 week to 4 weeks (Fig. 4D). In contrast, Csrp2^−/−^Apoe^−/−^ mice showed less collagen in the aneurysmal segments (Fig. 4E). Quantitative analysis confirmed that Csrp2^−/−^Apoe^−/−^ mice had significantly less collagen accumulation in the aneurysmal segments at 2 (Fig. 4F) and 4 weeks (Fig. 4G). In the normal arterial wall, type I and type III collagens are the main types in the media and adventitia [35–37], providing healthy aortic tissue with tensile strength [33]. Increased Col I synthesis was detected in the mouse aneurysm model [38] and Col III was found to be significantly increased in patient AAA samples [39]. We therefore, evaluated whether Col I and Col III levels were altered in the absence of CRP2. Col I immunostaining of abdominal aortic sections from saline infused mice for 2 weeks showed that Col I was expressed mainly in the adventitia and there were no apparent differences between Apoe^−/−^ and Csrp2^−/−^Apoe^−/−^ mice (Additional file 2: Fig. S3B). Western blot analysis of aortic proteins also showed that Col I expression level was not different between the two groups of mice. The low Col I level was likely because of aortic proteins were prepared from adventitia-stripped aortas. In contrast to Col I, Col III was detected predominantly in the media and the levels were much lower in the Csrp2^−/−^Apoe^−/−^ mouse aortic sections (Additional file 2: Fig. S3C). Western blot analysis of aortic proteins revealed that consistent with immunohistochemistry results, loss of CRP2 resulted in decreased baseline Col III (Additional file 2: Fig. S3C). Interestingly, 2 weeks after Ang II-infusion, Col III staining intensity was significantly elevated in Apoe^−/−^ compared to Csrp2^−/−^Apoe^−/−^ aneurysmal segments (Fig. 5A, B). Furthermore, Ang II significantly increased Col III expression levels in Apoe^−/−^ VSMCs while CRP2 deficiency led to a diminished Col III induction in Csrp2^−/−^Apoe^−/−^ VSMCs (Fig. 5C, D).

### Loss of CRP2 attenuates Ang II-induced Erk1/2 activation

**Fig. 6:**
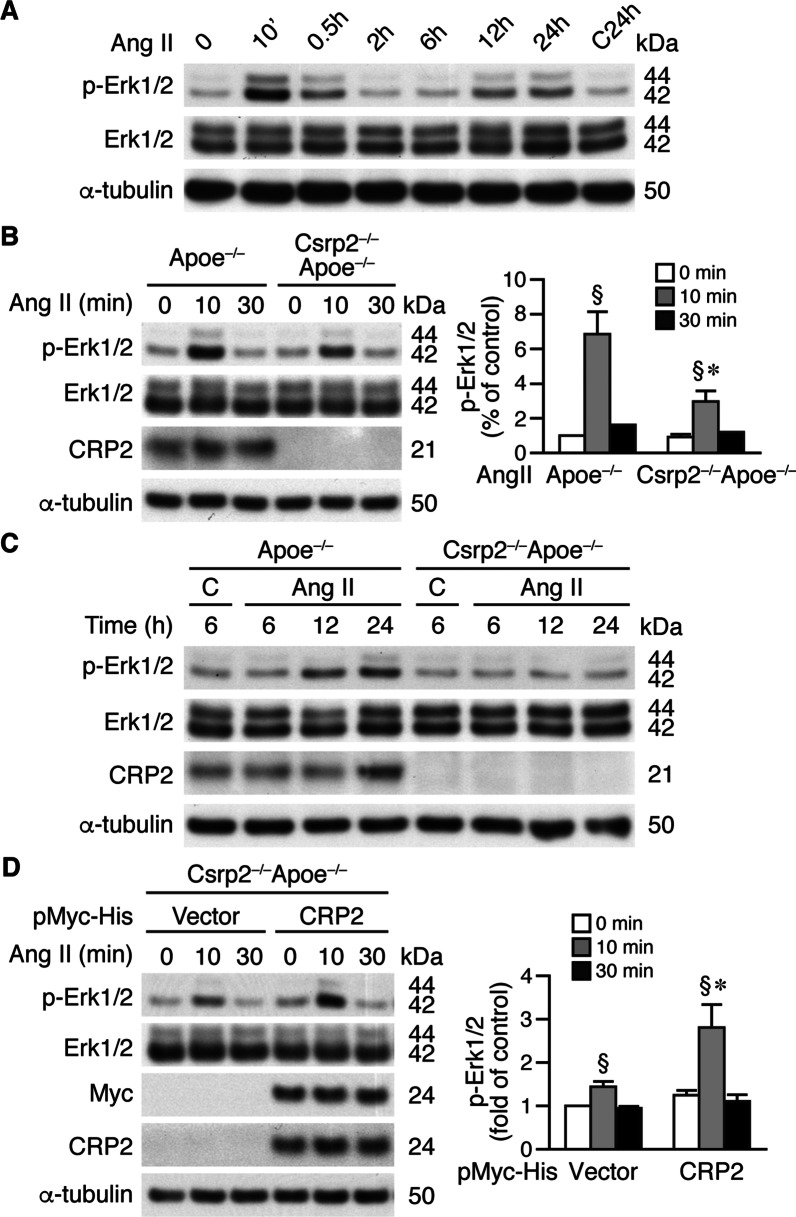
Loss of CRP2 attenuates Ang II-induced Erk1/2 activation. **A** VSMCs were treated with 10 µmole/L Ang II and total proteins prepared at different time points for Western blot analysis to detect phosphorylated and total Erk1/2, and α-tubulin as a loading control. **B** Left panel, Apoe^**−/−**^ and Csrp2^−/−^Apoe^−/−^ VSMCs were stimulated with Ang II and proteins harvested at 0, 10, and 30 min for Western blot analysis to detect phosphorylated and total Erk1/2, CRP2, and α-tubulin. A representative of 4 independent experiments is shown. Right panel, Quantitation of phospho-Erk1/2. Phosphorylated Erk1/2 level from Apoe^**−/−**^ VSMCs without Ang II treatment was set as 1. ^§^*P* < 0.05 compared with the respective baseline without Ang II; *P < 0.05 compared with the corresponding Apoe^−/−^ group. **C** Apoe^**−/−**^ and Csrp2^−/−^Apoe^−/−^ VSMCs were stimulated with Ang II and proteins harvested at 6, 12, and 24 h for Western blot analysis. **D** Left panel, Csrp2^−/−^Apoe^−/−^ VSMCs were transfected with pMyc-His vector or pMyc-His-CRP2 plasmid and treated with Ang II. Total proteins were then harvested at 0, 10, and 30 min for Western blot analysis to detect phosphorylated and total Erk1/2, CRP2, and Myc. A representative of 3 independent experiments is shown. Right panel, Quantitation of phospho-Erk1/2. Phosphorylated Erk1/2 level from pMyc-His-vector-transfected VSMCs at time 0 was set as 1. ^§^*P* < 0.05 compared with the respective baseline without Ang II; *P < 0.05 compared with the corresponding vector group

Our results indicate that CRP2 has an important role in controlling MMP2 and Col III expression, we next investigated the underlying molecular mechanisms. MAP kinase signaling pathways have been implicated in Ang II-induced AAA [40, 41]. We thus examined activation of MAP kinase signaling in VSMCs after Ang II treatment. Ang II induced activation of JNK at 10 min, to a similar degree in both Apoe^−/−^ and Csrp2^−/−^Apoe^−/−^ VSMCs (Additional file 2: Fig. S5). In comparison, Ang II did not activate p38 within the 30 min time period in VSMCs in either group. We next examined Erk1/2 activation in Apoe^−/−^ VSMCs after Ang II treatment. There was a biphasic activation of Erk1/2, first at 10–30 min, peaking at 10 min, and a second phase at 12–24 h (Fig. 6A). Evaluation of Erk1/2 phosphorylation in Apoe^−/−^ and Csrp2^−/−^Apoe^−/−^ VSMCs at the early (Fig. 6B) or late phase (Fig. 6C) revealed that Ang II-induced Erk1/2 activation at 10 min, 12 h, and 24 h was higher in Apoe^−/−^ than that in Csrp2^−/−^Apoe^−/−^ VSMCs (Fig. 6B, C), suggesting lack of CRP2 impaired Ang II-induced Erk1/2 activation. To rescue Erk1/2 activation, we transfected Csrp2^−/−^Apoe^−/−^ VSMCs with control or pMyc-His-CRP2 expression plasmid to reintroduce CRP2 expression and stimulated cells with Ang II. Western blot analysis showed that re-expression of CRP2 rescued Ang II-induced Erk1/2 phosphorylation (Fig. 6D).

### CRP2 regulates Ang II-induced MMP2 and col III via Erk1/2 signaling

**Fig. 7:**
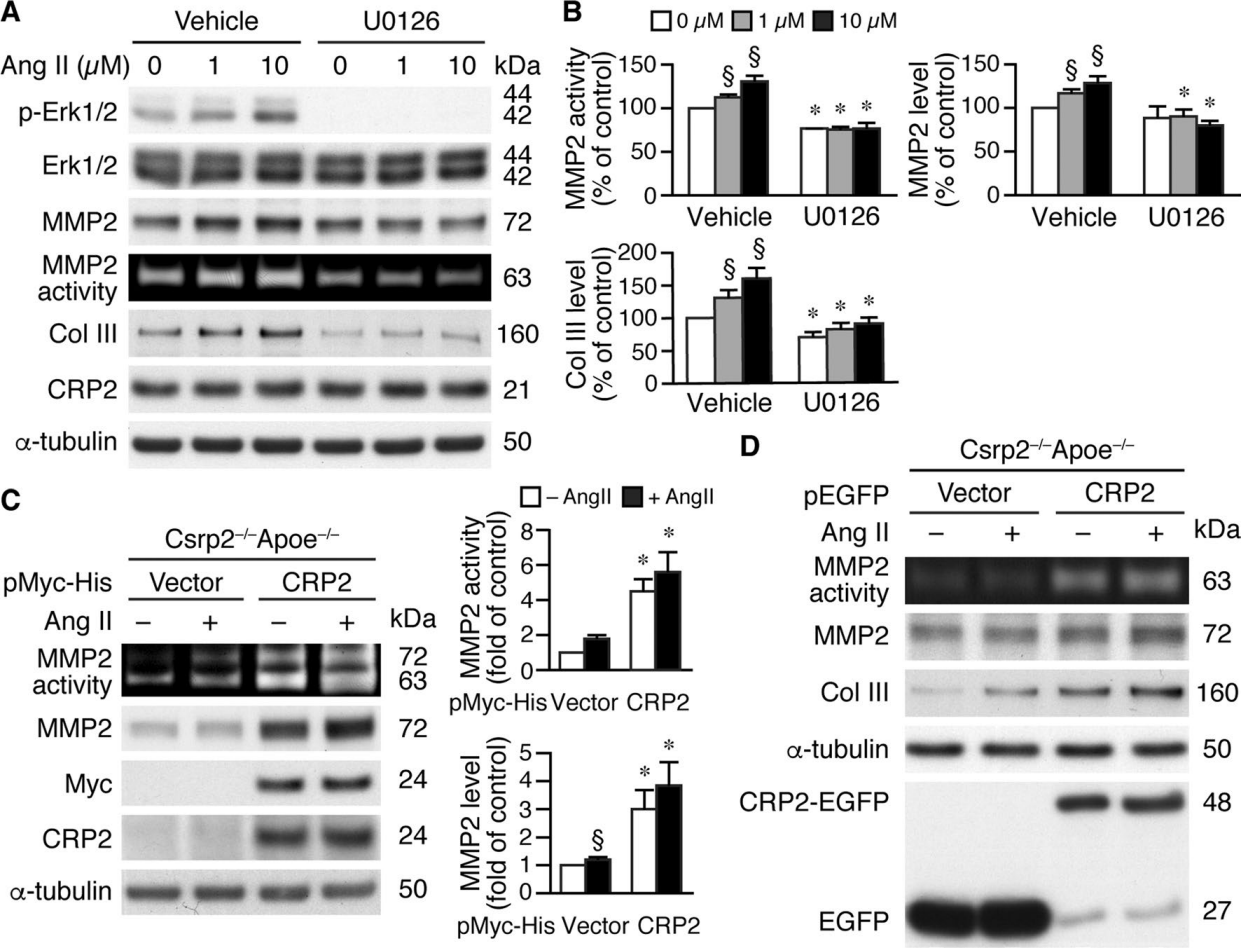
CRP2 regulates Ang II-induced MMP2 and Col III via Erk1/2 signaling. **A** Apoe^**−/−**^ VSMCs were pretreated with U0126 for 30 min before Ang II stimulation. Proteins or conditioned medium were prepared 24 h later. Western blot analysis was performed to detect Erk1/2 phosphorylation, total Erk1/2, CRP2, MMP2, and Col III expression. α-Tubulin was used to verify loading. Zymography was performed to measure MMP2 activity. A representative of 3–4 independent experiments is shown. **B** Quantitation of MMP2 activity (n = 4), MMP2 (n = 4) and Col III (n = 3) levels. The expression level of vehicle-treated, without Ang II stimulation was set as 100%. §*P* < 0.05 compared with the vehicle baseline without Ang II; *P < 0.05 compared with the corresponding vehicle group. **C** Left panel, Csrp2^−/−^Apoe^−/−^ VSMCs were transfected with pMyc-His or pMyc-His-CRP2 expression plasmids and treated with Ang II. Conditioned medium and total proteins were then collected 24 h later for zymography to determine MMP2 activity and protein expression (Myc, CRP2, and α-tubulin), respectively. A representative of 3 independent experiments is shown. Right panel, Quantitation of MMP2 activity and expression level. pMyc-His-vector-transfected MMP2 activity or expression level without Ang II stimulation was set as 1. n = 3 each group. §*P* < 0.05 compared with the vector baseline without Ang II; *P < 0.05 compared with the corresponding vector group. **D** Csrp2^−/−^Apoe^−/−^ VSMCs were transfected with pEGFP or pEGFP-CRP2 plasmids and treated with Ang II. After 24 h, conditioned medium was collected for zymography to determine MMP2 activity and total proteins prepared for Western blot analysis to detect expression levels of MMP2, Col III, CRP2-EGFP, and α-tubulin as a loading control

To determine whether Erk1/2 signaling controls expression levels and activity of MMP2, Col III, and CRP2, Apoe^−/−^ VSMCs were pretreated with Erk1/2 inhibitor U0126, stimulated with Ang II for 24 h, and total proteins prepared for Western blot analysis. U0126 blocked Ang II-mediated Erk1/2 activation, but did not affect CRP2 expression (Fig. 7A). U0126 attenuated Ang II-induced MMP2 protein expression and activity, and reduced Ang II-increased Col III levels (Fig. 7A, B), similar to the phenomena observed in Csrp2^−/−^Apoe^−/−^ VSMCs. Of note, without Ang II treatment, MMP2 activity and expression levels of MMP2 and Col III were reduced by U0126. We next re-expressed CRP2 in Csrp2^−/−^Apoe^−/−^ VSMCs using pMyc-His-CRP2 plasmid, treated transfected cells for 24 h with Ang II. Reintroduction of CRP2 in the double-knockout VSMCs rescued MMP2 activity and protein level either in the absence or presence of Ang II (Fig. 7C). To further independently support these findings, we transfected Csrp2^−/−^Apoe^−/−^ VSMCs with a different set of CRP2 expression plasmids, pEGFP-vector and pEGFP-CRP2. Indeed, compared with vector, expression of EGFP-CRP2 increased MMP2 activity and protein levels and Col III in these VSMCs either in the absence or presence of Ang II (Fig. 7D).

## Discussion

In this study, we identified a key role for CRP2 in mediating AAA formation. Loss of CRP2 attenuated Ang II-induced AAA incidence and severity, accompanied by preserved SM α-actin expression and reduced collagen deposition, MMP2 levels, aortic tensile strength, and blood pressure. Importantly, CRP2 deficiency not only maintained medial VSMC density, reduced basal MMP2 and Col III expression in VSMCs, but also mitigated Ang II-induced increases of these molecules via diminished Erk1/2 activation, thereby maintaining aortic ECM homeostasis and structural integrity and alleviating AAA development.

Medial VSMCs and their phenotypic modulation have been implicated in the development of aortic aneurysm [12, 42]. However, direct evidence for functional roles of contractile proteins in aortic aneurysm formation remained lacking until a recent study showing that the actin-binding, cytoskeletal protein SM22α could prevent aortic aneurysm formation by inhibiting VSMC phenotypic changes [43]. As CRP2 is highly and preferentially expressed in VSMCs, protects against arterial injury-induced intimal hyperplasia [20], and that both CRP2 and SM22α were identified as top differentially expressed genes in IAA SMC clusters [8], we initially hypothesized CRP2 may play a similar role as SM22α in reducing AAA formation. However, we found that CRP2 deficiency, unlike SM22α, attenuated AAA development. As AAA is characterized by degeneration of the elastic media, thinning and ballooning of the aortic wall whereas the lesions of stenosed arteries are mainly limited to the intimal layer of the arteries [21], CRP2 may play differential roles in AAA and occlusive vascular disease.

Interestingly, loss of CRP2 mitigated Ang II-increased collagen deposition in the aneurysmal aortic segments. Consistent with the concept that elevated levels of collagen result in increased arterial stiffness and compromised aortic compliance [44], the blood pressure of Apoe^−/−^ mice was significantly higher than that of Csrp2^−/−^Apoe^−/−^ mice following Ang II infusion. VSMCs are primarily responsible for the synthesis of ECM proteins and Col I and III are major constituents of the total collagen in the aorta [11]. While Col III provides normal aortic tissue with tensile strength [33], it was significantly increased in human AAA [39], indicating excessive Col III may correlate with development of aneurysm. Intriguingly, loss of CRP2 did not affect Col I expression; but resulted in lower baseline levels of Col III and attenuated Ang II-induced Col III in aneurysmal aorta and VSMCs. Moreover, lack of CRP2 diminished Ang II-increased aortic tensile strength, which could be due to decreases in Col III. These findings support a concept that although Col III is necessary for maintaining normal aortic structure and tensile strength, excessive Col III induced by Ang II may render aorta become stiff, predisposing to aneurysm formation and rupture. Conversely, attenuated Ang II induction of Col III due to CRP2 deficiency mitigates Ang II-induced exaggerated tensile strength, increased SBP, and aneurysm formation. Although it is recognized that hypertension is a risk factor for AAA formation [30], a prior report showed that Ang II infusion to hyperlipidemic mice induced AAA formation independent of blood pressure [45]. Supporting the concept that hypertension is not the only determinant for inducing AAA, we found that despite increased SBP, Apoe^−/−^ mice on a regular chow diet and infused with Ang II had a much lower AAA incidence (Additional file 2: Fig. S4). These findings indicate that both hypertension and high fat diet are necessary for promoting AAA in mice. Alternatively, it is possible that the attenuated increases of SBP in Csrp2^−/−^Apoe^−/−^ mice infused with Ang II may be a contributing factor to reduced arterial stiffness and AAA formation. Previous studies found similar Ang II-induced AAA formation in male Apoe^−/−^ mice fed either a low fat or high fat Western diet [46]. That study revealed that modest hypercholesterolemia augmented AAA, and progressive increases in hypercholesterolemia did not further enhance AAA. In addition, ApoB-containing lipoproteins contributed to the augmentation of Ang II-induced AAA in male mice [46]. In our study, high fat diet substantially increased AAA incidence in Apoe^−/−^ mice. The differences in our findings and those of Liu et al. could be related to the different diet employed. Our mice were fed a high fat diet containing 1.25% cholesterol [25], which is higher than the 0.15% used by Liu et al. [46]. Furthermore, despite the fat content of these diets was similar (20–21%), the source was different (cocoa butter and soybean oil vs. milk fat). Although we did not measure plasma lipoprotein profiles, different fat sources and higher cholesterol content could potentially explain the differences in our findings in our Apoe^−/−^ mice vs. the work of Liu et al. Subtle differences in mouse genetic background related to C57BL/6 substrains or housing could also contribute to these differences.

Another major finding of our study is that loss of CRP2 reduced basal and Ang II-induced MMP2 protein and activity in VSMCs and aorta. Increased synthesis of MMPs by aortic VSMCs contributes to the pathogenesis of AAA [11, 47]. In particular, single-cell transcriptomic profiling of VSMCs in Marfan syndrome aortic aneurysm revealed that MMP2 expression was significantly enriched in phenotypically modulated VSMCs, emphasizing a central role of MMP2 in ECM degradation and remodeling in aortic aneurysm [42]. These reports support and highlight the significance of reduced MMP2 activity in the absence of CRP2. Further confirming the role of CRP2 in regulating MMP2 and Col III expression, restoration of CRP2 expression in Csrp2^−/−^Apoe^−/−^ VSMCs rescued basal and Ang II-induced MMP2 (protein and activity) and Col III levels. Taken together, our results clearly establish that in response to Ang II stimulation, presence of CRP2 increases VSMC MMP2 and Col III expression, which in turn promotes AAA formation and development, whereas absence of CRP2 blocks increases of MMP2 and Col III induced by Ang II. Despite the established matrix-degrading function of MMP2 in AAA formation, Ang II-infused MMP2^−/−^ mice showed more severe dilation of the thoracic aorta and thoracic aortic aneurysm [48]. The study revealed that MMP2 has a dual role in ECM degradation and ECM synthesis. The authors postulated that the differential regional susceptibility of the aorta to aneurysm may be because thoracic aortas are more susceptible to impaired ECM synthesis whereas abdominal aortas are more vulnerable to aberrant ECM degradation [48].

Several studies have implicated Erk1/2 activation in the development of AAA. For example, Erk1/2 activation was enhanced in human AAA lesions and Ang II-infused mouse aneurysmal aortic media [40] whereas compounds that attenuated Ang II-induced AAA were found to block Erk1/2 activation [41, 49, 50]. Corroborating and supporting this notion, lack of CRP2 diminished Ang II-induced Erk1/2 activation while re-expression of CRP2 in Csrp2^−/−^Apoe^−/−^ VSMCs rescued Erk1/2 activation induced by Ang II. Furthermore, Erk1/2 inhibition by U0126 reduced Col III expression and MMP2 expression and activity. These results indicate that in response to Ang II stimulation, CRP2 regulates Erk1/2 activation and the downstream induction of MMP2 and Col III. It is not clear how CRP2 regulates Erk1/2 signaling; however, it is reasonable to speculate that the subtle cytoarchitectural alterations in the absence of the cytoskeletal CRP2 may affect the Erk1/2 cellular signaling pathway. This aspect of study will be worth pursuing in the future. Interestingly, the cytoskeletal protein SM22α reduces aortic aneurysm formation through the ROS/NF-κB pathway [43], indicating CRP2 and SM22α may control AAA formation via different signaling pathways. Our study revealed that CRP2 plays distinct roles in regulating VSMC functions under different disease settings. In contrast to its role in occlusive vascular disease by inhibiting VSMC migration into the intimal space through sequestering the scaffold protein p130Cas at focal adhesions [19], CRP2 mediated Ang II-activated Erk1/2 signaling pathways, thereby causing aberrant aortic ECM remodeling and AAA formation.

## Conclusions

Taken together, our data demonstrate that CRP2 has a critical role in the pathogenesis of AAA. In response to Ang II stimulation, loss of CRP2 maintains medial VSMC density and aortic ECM homeostasis, which is mediated via Erk1/2–Col III and MMP2 axis. Lack of CRP2 abrogates Ang II-induced Erk1/2 activation, subsequent induction of Col III and MMP2 levels, thereby attenuating adverse aortic remodeling and AAA formation. Our results suggest that targeting CRP2 will be beneficial in devising new therapeutic strategies against AAA.

## Supplementary information

**Additional file 1.** List of antibodies used

**Additional file 2: Fig. S1.** Total cholesterol level and aortic morphology in Apoe^−/−^ and Csrp2^−/−^Apoe^–/–^ mice. Apoe^−/−^ and Csrp2^−/−^Apoe^−/−^ mice were infused with saline or Ang II and fed a high-fat diet for 4 weeks. **A** Representative aortas from Apoe^−/−^ (n = 12) and Csrp2^−/−^Apoe^−/−^ (n = 13) mice infused with saline. **B** Total cholesterol levels from plasma of Apoe^−/−^ and Csrp2^−/−^Apoe^−/−^ mice infused with saline (n = 6 and 10, respectively) or Ang II (n = 6 and 5, respectively). **C** Histological analysis of abdominal aortas from Apoe^−/−^ and Csrp2^−/−^Apoe^−/−^ mice infused with saline. Aortic sections were stained with H&E, SM α-actin for smooth muscle cells, and Verhoeff’s stain elastin to delineate elastin layers. **Fig. S2.** Baseline ROS level and MMP activity in the aortic wall of Apoe^−/−^ and Csrp2^−/−^Apoe^−/−^ mice. Mice were infused with saline for 2 weeks and abdominal aortas harvested for analysis. **A** DHE staining (red fluorescence) was performed to assess ROS level on abdominal aortic sections. **B** In situ zymography was performed to measure MMP activity (green fluorescence) on abdominal aortic sections. Med, media; Lu, lumen. **Fig. S3.** Expression of MMP9, collagen I, and collagen III in the mouse aorta. Apoe^−/−^ and Csrp2^−/−^Apoe^−/−^ mice were infused with saline for 2 weeks and abdominal aortas harvested for immunohistochemistry. **A** Abdominal aortic sections were stained with MMP9 antibody. Aneurysmal aortic sections were used as positive control (right panel). Arrows indicate positive staining of MMP9 in the infiltrated immune cells. **B** Abdominal aortic sections were stained with collagen I (Col I) antibody (left two panels). Right panel, total proteins were prepared from Apoe^−/−^ and Csrp2^−/−^Apoe^−/−^ mouse aortas for Western blot analysis to detect Col I and CRP2 expression, and α-tubulin was used as a loading control. **C** Abdominal aortic sections were stained with collagen III (Col III) antibody (left two panels). Right panel, total proteins were prepared from Apoe^−/−^ and Csrp2^−/−^Apoe^−/−^ mouse aortas for Western blot analysis to detect Col III and CRP2 expression, and α-tubulin was used as a loading control. **Fig. S4**. CRP2 modulates AngII-induced AAA incidence and blood pressure. Mice were infused with saline or AngII for 4 weeks while on chow diet and aortas harvested for examination of aneurysm formation. **A** Apoe^−/−^ and **B** Csrp2^−/−^Apoe^−/−^ mice were infused with Ang II for 4 weeks. White arrow, abdominal aneurysm. **C** Saline infusion did not induce AAA formation in Apoe^−/−^ (n = 3) or Csrp2^−/−^Apoe^−/−^ (n = 2) mice. Ang II infusion for 4 weeks induced AAA in 33% of Apoe^−/−^ mice (n = 9) while no AAA was observed in Csrp2^−/−^Apoe^−/−^ (n = 7) mice. **D** Systolic blood pressure of Apoe^−/−^ (n = 4) and Csrp2^−/−^Apoe^−/−^ (n = 4) mice was measured at baseline and at different days (7, 10, 14, and 21 d) following Ang II infusion. *P < 0.05 vs. Csrp2^−/−^Apoe^−/−^ mice. **Fig. S5.** Effect of Ang II on JNK and p38 activation. Apoe^−/−^ and Csrp2^−/−^Apoe^−/−^ VSMCs were treated with 10 µmol/L Ang II for different period of time. As a positive control, Apoe^−/−^ VSMCs were treated with 10 ng/mL IL-1β for 10 min. Total proteins were prepared for Western blot analysis to detect phosphorylated and total JNK and p38, and CRP2. The blots were subsequently hybridized with α-tubulin as a loading control

## Data Availability

All data generated or analyzed during this study are included in this published article and its Additional files.

## Abbreviations

AAA: Abdominal aortic aneurysm
Ang II: Angiotensin II
AOD: Aortic occlusive disease
Col I: Collagen type I
Col III: Collagen type III
CRP2: Cysteine-rich protein 2
Csrp2: Cysteine and glycine rich protein 2 (gene name for CRP2)
ECM: Extracellular matrix
EGFP: Enhanced green fluorescent protein
HRP: Horseradish peroxidase
IRA: Infrarenal aorta
MMP2: matrix metalloproteinase 2
MMP9: matrix metalloproteinase 9
ROS: Reactive oxygen species
SBP: Systolic blood pressure
SM: Smooth muscle
SRA: Suprarenal aorta
VSMCs: Vascular smooth muscle cells
