# Supplementary material for: Cysteine-rich protein 2 deficiency attenuates angiotensin II-induced abdominal aortic aneurysm formation in mice
Source: J Biomed Sci. 2022 Apr 12;29:25. doi: 10.1186/s12929-022-00808-z (PMC9004090; doi:10.1186/s12929-022-00808-z)
Supplement: Supplementary file 1 — Additional file 1. List of antibodies used [file 12929_2022_808_MOESM1_ESM.pdf]

Western blot analysis

Primary antibody	Vendor	Cat. Number	Secondary antibody	Vendor	Cat. Number
CRP2-(81-97)	GenScript	Custom-made	Donkey anti-goat	Jackson	705035003
Col I	Abcam	ab88147	Goat anti-Mouse	Millipore	AP124P
Col III	Abcam	ab7778	Goat anti-Rabbit	Thermo	31460
Erk1/2	Cell Signaling	9102	Goat anti-Rabbit	Thermo	31460
p-Erk1/2	Cell Signaling	9106	Goat anti-Mouse	Millipore	AP124P
JNK	Cell Signaling	9252	Goat anti-Rabbit	Thermo	31460
p-JNK	Cell Signaling	9251	Goat anti-Rabbit	Thermo	31460
MMP2	Abcam	ab37150	Goat anti-Rabbit	Thermo	31460
MMP9	R&D Systems	AF909	Donkey anti-goat	Jackson	705035003
Myc	Cell Signaling	2278	Goat anti-Rabbit	Thermo	31460
p38MAPK	Cell Signaling	9212	Goat anti-Rabbit	Thermo	31460
p-p38MAPK	Cell Signaling	9211	Goat anti-Rabbit	Thermo	31460
Pan-actin	Millipore	MAB1501	Goat anti-Mouse	Millipore	AP124P
α-Tubulin	Cell Signaling	3873	Goat anti-Mouse	Millipore	AP124P

Immunohistochemistry

Primary antibody	Vendor	Cat. Number	Secondary antibody	Vendor	Cat. Number
CRP2-(81-97)	GenScript	Custom-made	Donkey anti-goat	Santa cruz	sc2020
Col I	Abcam	ab88147	Goat anti-Mouse	DAKO	K4001
Col III	Abcam	ab7778	Goat anti-Rabbit	DAKO	K4003
MMP2	Abcam	ab37150	Goat anti-Rabbit	DAKO	K4003
MMP9	Abcam	ab38898	Goat anti-Rabbit	DAKO	K4003
SM α-actin	Sigma-Aldrich	A5528	Goat anti-Mouse	DAKO	K4001
