# Supplementary material for: Cysteine-rich protein 2 deficiency attenuates angiotensin II-induced abdominal aortic aneurysm formation in mice
Source: J Biomed Sci. 2022 Apr 12;29:25. doi: 10.1186/s12929-022-00808-z (PMC9004090; doi:10.1186/s12929-022-00808-z)
Supplement: Supplementary file 2 — Additional file 2: Fig. S1. Total cholesterol level and aortic morphology in Apoe−/− and Csrp2−/−Apoe–/– mice. Apoe−/− and Csrp2−/−Apoe−/− mice were infused with saline or Ang II and fed a high-fat diet for 4 weeks. A Representative aortas from Apoe−/− (n = 12) and Csrp2−/−Apoe−/− (n = 13) mice infused with saline. B Total cholesterol levels from plasma of Apoe−/− and Csrp2−/−Apoe−/− mice infused with saline (n = 6 and 10, respectively) or Ang II (n = 6 and 5, respectively). C Histological analysis of abdominal aortas from Apoe−/− and Csrp2−/−Apoe−/− mice infused with saline. Aortic sections were stained with H&E, SM α-actin for smooth muscle cells, and Verhoeff’s stain elastin to delineate elastin layers. Fig. S2. Baseline ROS level and MMP activity in the aortic wall of Apoe−/− and Csrp2−/−Apoe−/− mice. Mice were infused with saline for 2 weeks and abdominal aortas harvested for analysis. A DHE staining (red fluorescence) was performed to assess ROS level on abdominal aortic sections. B In situ zymography was performed to measure MMP activity (green fluorescence) on abdominal aortic sections. Med, media; Lu, lumen. Fig. S3. Expression of MMP9, collagen I, and collagen III in the mouse aorta. Apoe−/− and Csrp2−/−Apoe−/− mice were infused with saline for 2 weeks and abdominal aortas harvested for immunohistochemistry. A Abdominal aortic sections were stained with MMP9 antibody. Aneurysmal aortic sections were used as positive control (right panel). Arrows indicate positive staining of MMP9 in the infiltrated immune cells. B Abdominal aortic sections were stained with collagen I (Col I) antibody (left two panels). Right panel, total proteins were prepared from Apoe−/− and Csrp2−/−Apoe−/− mouse aortas for Western blot analysis to detect Col I and CRP2 expression, and α-tubulin was used as a loading control. C Abdominal aortic sections were stained with collagen III (Col III) antibody (left two panels). Right panel, total proteins were prepared from Apoe−/− and Csrp2−/ [file 12929_2022_808_MOESM2_ESM.pdf]

Figure S1

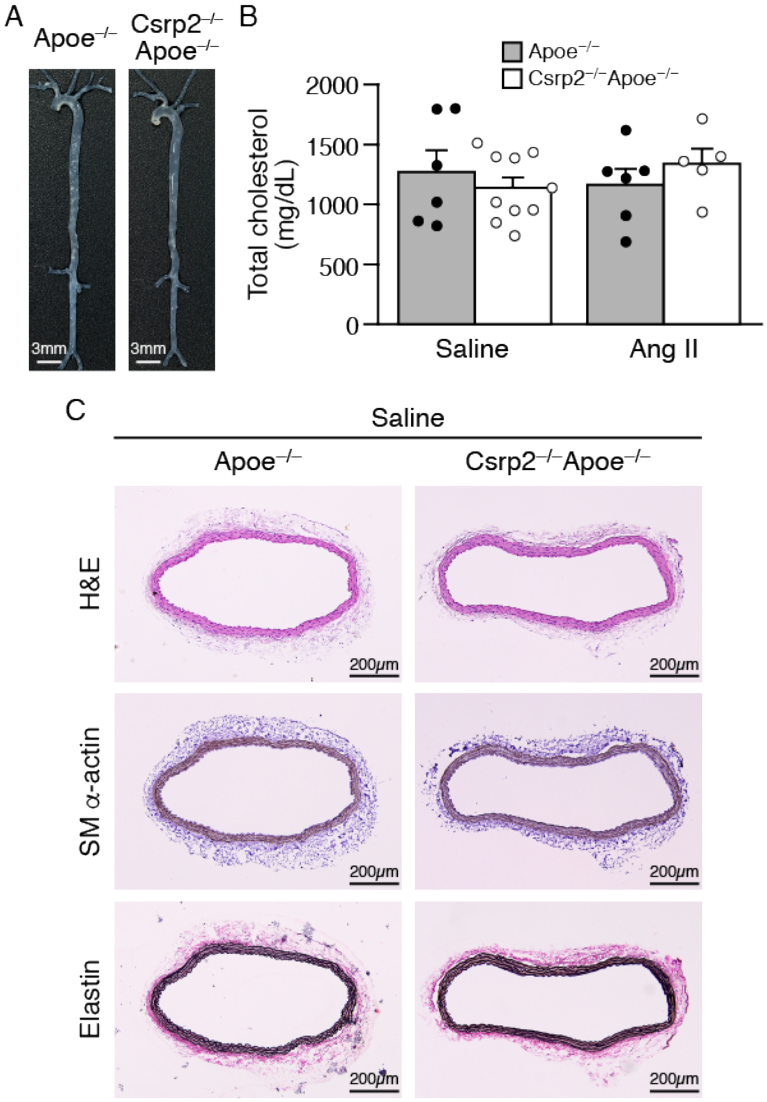

Fig. S1. Total cholesterol level and aortic morphology in Apoe$^{-/-}$ and Csrp2$^{-/-}$Apoe$^{-/-}$ mice. Apoe$^{-/-}$ and Csrp2$^{-/-}$Apoe$^{-/-}$ mice were infused with saline or Ang II and fed a high-fat diet for 4 weeks. (A) Representative aortas from Apoe$^{-/-}$ (n=12) and Csrp2$^{-/-}$Apoe$^{-/-}$ (n=13) mice infused with saline. (B) Total cholesterol levels from plasma of Apoe$^{-/-}$ and Csrp2$^{-/-}$Apoe$^{-/-}$ mice infused with saline (n=6 and 10, respectively) or Ang II (n= 6 and 5, respectively). (C) Histological analysis of abdominal aortas from Apoe$^{-/-}$ and Csrp2$^{-/-}$Apoe$^{-/-}$ mice infused with saline. Aortic sections were stained with H&E, SM α-actin for smooth muscle cells, and Verhoeff's stain to delineate elastin layers.

Figure S2

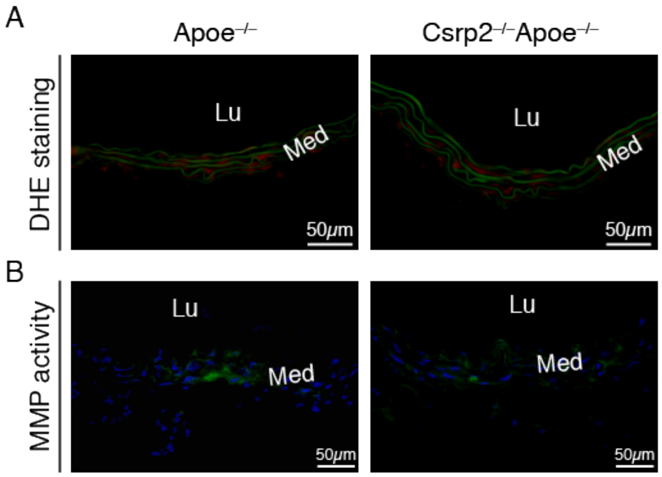

Fig. S2. Baseline ROS level and MMP activity in the aortic wall of $Apoe^{-/-}$ and $Csrp2^{-/-}Apoe^{-/-}$ mice. Mice were infused with saline for 2 weeks and abdominal aortas harvested for analysis. (A) DHE staining (red fluorescence) was performed to assess ROS level on abdominal aortic sections. (B) *In situ* zymography was performed to measure MMP activity (green fluorescence) on abdominal aortic sections. Med, media; Lu, lumen.

Figure S3

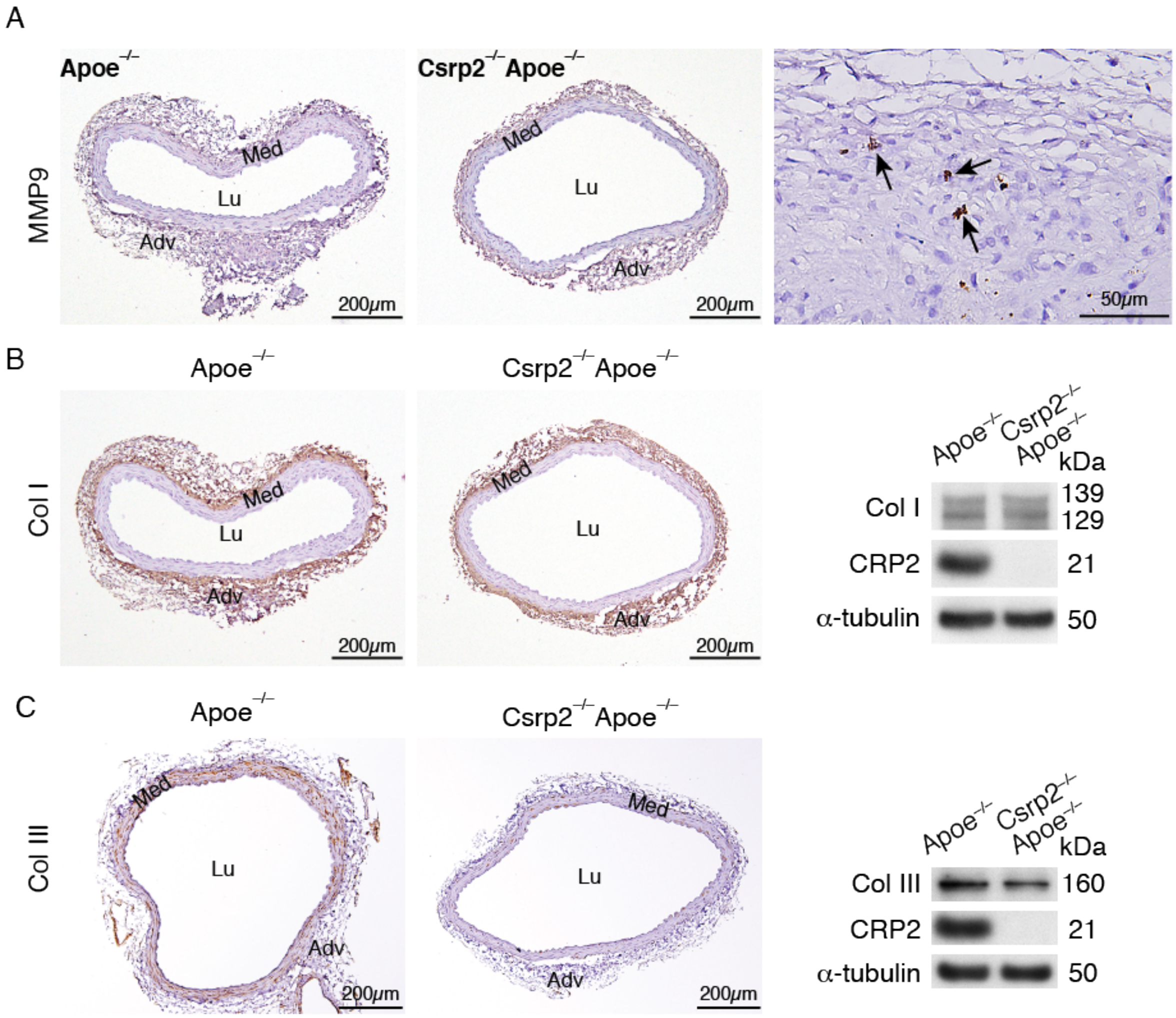

Fig. S3. Expression of MMP9, collagen I, and collagen III in the mouse aorta. Apoe$^{-/-}$ and Csrp2$^{-/-}$Apoe$^{-/-}$ mice were infused with saline for 2 weeks and abdominal aortas harvested for immunohistochemistry. (A) Abdominal aortic sections were stained with MMP9 antibody. Aneurysmal aortic sections were used as positive control (right panel). Arrows indicate positive staining of MMP9 in the infiltrated immune cells. (B) Abdominal aortic sections were stained with collagen I (Col I) antibody (left two panels). Right panel, total proteins were prepared from Apoe$^{-/-}$ and Csrp2$^{-/-}$Apoe$^{-/-}$ mouse aortas for Western blot analysis to detect Col I and CRP2 expression, and α-tubulin was used as a loading control. (C) Abdominal aortic sections were stained with collagen III (Col III) antibody (left two panels). Right panel, total proteins were prepared from Apoe$^{-/-}$ and Csrp2$^{-/-}$Apoe$^{-/-}$ mouse aortas for Western blot analysis to detect Col III and CRP2 expression, and α-tubulin was used as a loading control.

Figure S4

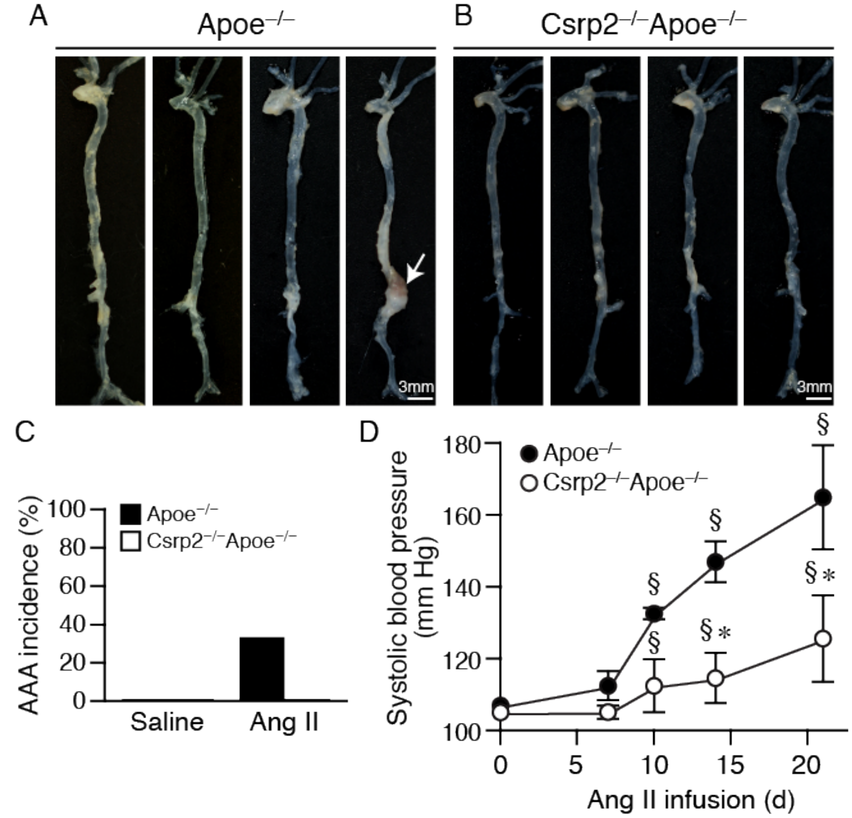

Fig. S4. CRP2 modulates AngII-induced AAA incidence and blood pressure. Mice were infused with saline or AngII for 4 weeks while on chow diet and aortas harvested for examination of aneurysm formation. (A) $Apoe^{-/-}$ and (B) $Csrp2^{-/-}Apoe^{-/-}$ mice were infused with Ang II for 4 weeks. White arrow, abdominal aneurysm. (C) Saline infusion did not induce AAA formation in $Apoe^{-/-}$ (n=3) or $Csrp2^{-/-}Apoe^{-/-}$ (n=2) mice. Ang II infusion for 4 weeks induced AAA in 33% of $Apoe^{-/-}$ mice (n=9) while no AAA was observed in $Csrp2^{-/-}Apoe^{-/-}$ (n=7) mice. (D) Systolic blood pressure of $Apoe^{-/-}$ (n=4) and $Csrp2^{-/-}Apoe^{-/-}$ (n=4) mice was measured at baseline and at different days (7, 10, 14, and 21 d) following Ang II infusion. §$P<0.05$ compared with the respective baseline at d 0. *$P<0.05$ compared with the corresponding $Apoe^{-/-}$ group.

Figure S5

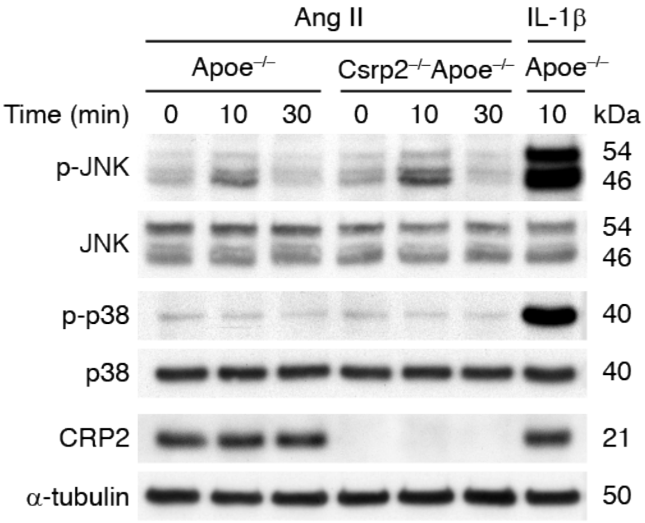

Fig. S5. Effect of Ang II on JNK and p38 activation. Apoe$^{-/-}$ and Csrp2$^{-/-}$Apoe$^{-/-}$ VSMCs were treated with 10 μmole/L Ang II for different period of time. As a positive control, Apoe$^{-/-}$ VSMCs were treated with 10 ng/mL IL-1β for 10 min. Total proteins were prepared for Western blot analysis to detect phosphorylated and total JNK and p38, and CRP2. The blots were subsequently hybridized with α-tubulin as a loading control.
